# Monitoring climate change vulnerability in the Himalayas

**DOI:** 10.1007/s13280-024-02066-9

**Published:** 2024-08-31

**Authors:** Ishfaq Hussain Malik, James D. Ford

**Affiliations:** 1 School of Geography, University of Leeds, Leeds, LS2 9JT UK; 2 Priestley Centre for Climate Futures, University of Leeds, Leeds, LS2 9JT UK

**Keywords:** Climate change, Kashmir Himalayas, Longitudinal assessment, Monitoring, Re-study, Vulnerability

## Abstract

Longitudinal assessment of climate vulnerability is essential for understanding the complex factors affecting how people experience and respond to climate change. We report on the first longitudinal assessment of climate vulnerability in the Himalayan region, exploring the evolving landscape, perceptions, and experiences of communities of climate change impacts, vulnerability, and adaptation in Kashmir over an 8-year period from 2017 to 2024. We provide the Himalayan Re-study Framework (HRF) to monitor, characterise, and conceptualise climate change in the Himalayas. Utilising mixed methods, we showcase how climate change is affecting social, economic, political, and environmental dimensions, examining how the impacts of climate change and vulnerability evolve over time, shaping and reshaping how climate risks are experienced and responded to by communities. Our analysis reveals a nuanced understanding of vulnerability, highlighting the impact on communities’ livelihoods and water security, differential impacts on marginalised communities, and the gendered nature of climate change. We examine how certain sections of the population face marginalisation, discrimination, and racism, and how climate change exacerbates these challenges. Kashmir’s vulnerability to climate change extends beyond environmental factors, intertwining with culture, livelihoods, social dynamics, and politics. Climate change continues to compete for attention with immediate political and socio-economic challenges, highlighting the need for integrated approaches to address both environmental and societal issues in Kashmir.

## Introduction

The Himalayas, known as the ‘Roof of the World’, provide an ideal laboratory for studying the wide-ranging effects of climate change due to their complex socio-political dynamics and fragile ecosystems. With some of the poorest communities and most densely populated cities in the world, diverse climate zones, and ecosystem services benefiting billions of people, the region is experiencing significant climate change, with rising temperatures, changing precipitation patterns, rapid melting of snow and glaciers, and increasing disaster risk (Diksha et al. 2022; Chauhan et al. 2023; Dhital et al. 2023; ICIMOD 2024). The region is experiencing a faster rate of warming than the global average, ranging from 0.15 to 0.60 °C per decade, with implications for water supply and power generation (Singh and Gumber 2018; Mir et al. 2021; IPCC 2023). This has led to a range of ecological and societal impacts on nearly 240 million people living in the Himalayas and about 2 billion people depending on the Himalayan resources, particularly vulnerable communities with strong reliance on the environment, leading to challenges in adaptation, livelihoods, agriculture, water availability, and ways of life (Dhungana et al. 2020; Negi et al. 2021; Verma 2023; ICIMOD 2024; Malik and Ford 2024; Malik 2024). These changes are expected to continue, with increases in temperature and precipitation changes (Talchabhadel and Karki 2019) projected to affect 303.63 million people in 2030, especially Indigenous communities who are particularly susceptible, with their well-being and livelihoods at risk (Negi et al. 2021; ICIMOD, 2024).

The Kashmir region in the North-western Himalayas is experiencing significant impacts of climate change. Rising temperatures and decreasing precipitation, and a substantial increase in mean maximum and minimum temperatures, reduction in glacier mass and volume, as well as the increasing frequency and severity of extreme weather events have been observed, leading to changes in the region's economic, social, and environmental landscape (Mahdi et al. 2021; Ahsan et al. 2022; Lone et al. 2022). Kashmir is vulnerable to disasters like floods, earthquakes, landslides, and avalanches and has experienced an increasing number of flooding events in recent years (Malik 2022a, 2022b; Malik and Hashmi 2022). Communities are facing significant impacts of climate change, including water scarcity, loss of biodiversity and local food varieties, wetlands, and water resources, and shifts in ecosystem boundaries, impacts on agriculture and agricultural practices, and livelihood loss (Ahanger and Lone 2018; Lone et al. 2020; Mir and Batool 2022). Local perceptions of these changes are largely consistent with scientific evidence (Ishtiyak et al. 2016; Shafiq et al. 2019).

A range of studies have explored climate change impacts in different parts of the Himalayas, including the Kashmir Valley, examining glacial recession, altered snow cover, thawing permafrost, land use changes, hydrological changes related to cryosphere degradation, and adaptation actions in agriculture and water-related sectors (Barua et al. 2014; Mishra et al. 2014; Rashid et al. 2015; Shafiq et al. 2019; McDowell et al. 2019; Mahdi et al. 2021; Lone et al. 2022; Ahmad et al. 2024). This work has mostly been conducted within the physical sciences. There is limited research on the human dimensions of climate change which document the experiences of communities in the Kashmir Himalayas or examine changes over multiple years, how these changes have evolved over time and affect communities, and how these changes are perceived over the years.

This paper reports on the first longitudinal *re-study* of climate vulnerability in the Kashmir Himalayas (and also more broadly, the Himalayan region), examining trends over the period 2017 to 2024. Building upon the work of Ford et al. (2013), Fawcett et al. (2017) and Naylor et al. (2021), the study employs a longitudinal assessment, monitoring the impacts of climate change and community vulnerability and how they change over time, shaping and reshaping how climate risks are experienced and responded to by communities. Specifically, the study tracks the experiences and perceptions of community members over an eight-year period to: (i) identify and characterise if/how vulnerability is changing, (ii) document coping mechanisms and adaptations being employed, (iii) examine the success of coping mechanisms and adaptations and if/how they have changed over time, and (iv) assess differences between social groups over time. The approach is grounded by repeat interviews with the same individuals over an 8-year period, where the same questions are asked and then compared to previous interviews, allowing us to trace key drivers and trends in vulnerability.

We call this approach the Himalayan Re-study Framework (HRF). This framework has global relevance and is particularly important in the Himalayas because they are highly susceptible to climate impacts and climate-related disasters. The framework involves regular year-by-year and/or season-by-season monitoring, regular or irregular monitoring after a few years or seasons, or monitoring after an interval of several years with the same participants or participants with similar demographic characteristics to analyse temporal changes. To monitor vulnerability to climate change, it is imperative to track these changes and events not only from the perspective of the physical sciences but also from the social sciences, as these changes affect directly or indirectly billions of people’s economic, social, political, and environmental landscapes. The HRF encapsulates the periodically cyclical process of re-studying climate vulnerability processes and signifies an ongoing process of observation, recording, and analysis to address the evolving challenges posed by climate change in the region, inform effective adaptation strategies, and enhance resilience in Himalayan communities.

## Materials and methods

### Conceptual approach and methodology

Vulnerability is the degree to which a system or community is susceptible to, or unable to cope with, the adverse effects of climate change (IPCC 2007). Within this study, we conceptualise vulnerability as a function of exposure-sensitivity and adaptive capacity (IPCC 2014; Estoque et al. 2023). *Exposure-sensitivity* captures the extent to which a system or population is susceptible to the impacts of climate change and *adaptive capacity* captures the ability to address risks and cope with consequences, and are influenced by factors like livelihoods, equity, resources, social networks, and technological adaptation (Ford and Smit 2004; IPCC 2007). Vulnerability is affected by social, economic, environmental, and political factors, including power dynamics, inequality, and institutions that create and perpetuate vulnerability (Taylor 2013; Barnett 2020).

This research adopts a vulnerability approach that builds on Archer et al. (2017), Fawcett et al. (2017), and Naylor et al. (2021), that focuses on the dynamic nature of vulnerability that requires continuous monitoring to document, characterise, and evaluate its complexity. Complex adaptive systems theory is increasingly utilised in studies addressing social–ecological phenomena. It describes self-organised components whose interactions lead to emergent behaviours. This framework, emphasising time and system structure, offers insights into vulnerability dynamics arising from climate–society interactions and contextualises climate change within social and ecological dimensions and socio-economic drivers such as marginalisation, inequality, and exploitation (Ford et al. 2013; Ribot 2014; Wang et al. 2014; Barnett 2020; Naylor et al. 2021). To understand vulnerability in the current study, a mixed-methods methodology is employed to account for dynamism and cross-component interactions while considering socio-economic, cultural, and political conditions.

The study draws upon a re-study approach (e.g. Fawcett et al. (2017), Naylor et al. (2020, 2021)), with repeated observation of human–environment interactions over extended periods of time crucial for comprehending the dynamics of vulnerability. To capture this dynamism, our study adopts a longitudinal approach to analyse continuity and change over an 8-year period, conducting repeated assessments of vulnerability. By doing so, it helps reduce recall bias and provides valuable insights into the environmental and human factors that impact vulnerability. Longitudinal studies come in various formats like: (a) Continuous Research: This involves studying the same geography over several years without interruption; (b) Periodic Re-studies: Researchers revisit the same themes at regular or irregular intervals; and (c) Returning After a Lengthy Interval: In this case, the study involves returning to a specific context after a considerable amount of time has elapsed (Young et al. 1991; Fawcett et al. 2017; Archer et al. 2017). Our study falls into the first category, where we conducted the original study in 2017, then revisited it in subsequent years until 2024. We have revisited Kashmir to examine the same themes over an eight-year period after the original study.

### Study area

**Fig. 1:**
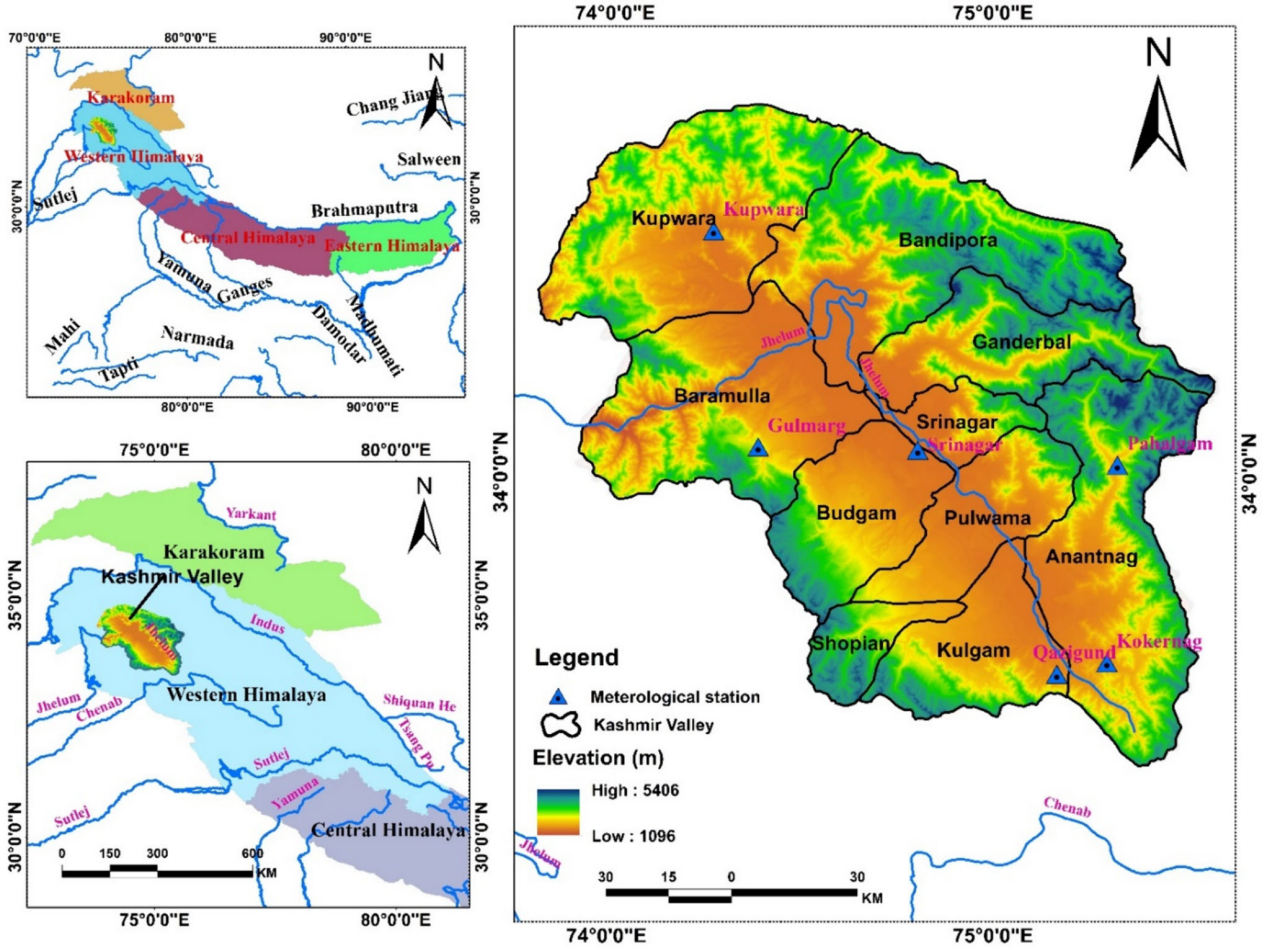
Study area map showing the location of the Kashmir Valley, situated in the Northwestern part of the Himalayas, along with its districts and rivers

Kashmir Valley is a Himalayan region lying in the North-Western part of the Himalayas (32° 20′-34° 50′N and 73° 55′-75° 35′E) (Fig. 1). The region is of immense geopolitical significance and is a critical source of conflict between India, Pakistan, and China. It has an area of 15,220km^2^ and a population of 7.5 million (Malik and Hashmi 2021; Malik 2022c; Wani and Malik 2023). Kashmir’s significance in studying climate change lies in its unique physical, political, and cultural geography, socio-economic dependence on agriculture and horticulture, and the impacts of global climate trends. The region’s retreating glaciers affect water availability in the streams and rivers, impacting agriculture, irrigation, and livelihoods. Changing land use patterns, economic vulnerability, and extreme weather events and dependence on natural resources underscore the need to understand Kashmir’s climate change dynamics.

### Methods

#### Data collection

This study employed a mixed-methods approach, integrating qualitative and quantitative data to provide a comprehensive overview of climate change responses, focusing on the Kashmiri community in general (with a special focus on Gujjar, Bakarwal, and Waatal communities) in Anantnag, Bandipora, Baramulla, Budgam, Ganderbal, Kulgam, Kupwara, Pulwama, Shopian, and Srinagar, where the interviews were conducted. Qualitative data were collected through semi-structured interviews and focus groups with stakeholders, including local communities, policymakers, journalists, academicians, and climate experts, as well as through ethnographic fieldwork, participatory mapping, and storytelling to gain a multi-perspective understanding of climate vulnerability processes. The fieldwork and interviews were conducted between 2017 and 2024 by the lead author, who is from the region and began the research while based at the Aligarh Muslim University, India, and continued at the University of Leeds, UK.

We used the same methods as the original baseline study, for which the fieldwork was conducted in 2017. Semi-structured interviews (*n* = 50; 5 each from ten locations) and focus groups (*n* = 10; 1 each from ten locations) were conducted in 2017. Additionally, semi-structured interviews (*n* = 30; 3 each from ten locations) and focus groups (*n* = 10; 1 each from ten locations) were conducted each year from 2018 to 2023. In 2024, semi-structured interviews (*n* = 20; 2 each from ten locations) and focus groups (*n* = 3) were conducted. The study also includes sixteen interviews with experts (2 each year). A total of 266 semi-structured interviews and 73 focus groups were conducted over an eight-year period. Most of the participants consisted of those that were involved in the original study, but due to the unavailability and passing away of some participants, similar types of participants were interviewed in subsequent years with similar demographic characteristics like age, livelihoods, and gender.

A purposive sampling technique was utilised to select participants from the Kashmiri community. Participants were identified based on their relevance to the research topic and their ability to provide valuable insights by the lead researcher, who is from the region and has nearly a decade of experience conducting research in Kashmir. Key stakeholders involved in local communities and their representatives, governmental officials, and experts in the fields of climate change and disaster management were included in the sample.

Semi-structured interviews were conducted through a series of in-depth interviews with the selected participants to collect in-depth information about their perspectives on vulnerability dynamics in Kashmir over the given time frame. The interviews were guided by a predetermined set of questions that were the same across the study years to enable comparison over time. The interviews were audio-recorded with the consent of the participants and subsequently transcribed for analysis. Focus groups were organised with local communities, their representatives, and key stakeholders.

Ethnographic research involved extensive participation and observation in community activities to gather nuanced insights into the lived experiences and adaptive practices in the face of climate change. It involved the researcher interacting with the local community and actively participating in their daily experiences and routines. This method helped to gain a deeper understanding of the sense of place and contextual factors contributing to vulnerability in Kashmir. A diary of these observations was kept throughout the years to note down the experiences and changes.

For the purposes of the study, we identified extreme weather events, changes in weather patterns, livelihoods, gendered nature, socio-economic conditions, access to basic services, geographic location, housing quality, political stability, adaptation initiatives, traditional knowledge, disaster preparedness, and capacity building indicators, which were used to analyse the key components of vulnerability, i.e. exposure-sensitivity and adaptive capacity (Table 1). The selection of indicators was determined based on their relevance to the specific conditions and characteristics of the Himalayan region. The index assigned to each category ranged from 0 to 10, with higher numbers indicating a higher level of vulnerability or impact. For uniformity and comparability between categories, all indicators and data sets were normalised on a scale from 0 to 10. To show the general trend in the observed changes, data from each category were layered to demonstrate their cumulative impact over time.

**Table 1:** ‘Selection of Indicators,’ listing the components analysed, the corresponding indicators selected, and the methods used for collecting each indicator

Component	Indicator	Indicator collection method
Exposure-sensitivity	Extreme weather events and disasters	Ethnographic studies, semi-structured interviews, community consultations, and focus group discussions
	Changes in temperature and precipitation patterns	Community perception, meteorological data, field observations, and historical records
	Changes in livelihoods and agricultural practices	Semi-structured interviews, focus group discussions with farmers, field diary, personal experience
	Gendered impacts	Gender-disaggregated interviews, and semi-structured interviews with communities and journalists
	Water scarcity, availability, and water resource changes	Semi-structured interviews and participant observation
	Displacement and migration patterns	Semi-structured interviews
	Socio-economic inequalities	Social network analysis, key informant interviews, semi-structured interviews
	Access to basic services (water supply, sanitation, healthcare, and education)	Health facility surveys, census data analysis, and semi-structured interviews
	Location (proximity to hazards, e.g. floodplains)	Participatory mapping, storytelling
	Housing quality: Kacha house (made of mud, bricks, and grass) and Pakka house (concrete houses made of cement, iron, and bricks)	Participant observation, semi-structured interviews
	Political stability	Participant observation, semi-structured interviews, focus group discussions, and key informant interviews
Adaptive capacity	Climate change adaptation initiatives	Community consultations, participatory workshops
	Sustainable livelihood diversification	Livelihood surveys, focus group discussions, semi-structured interviews, interviews of farmers
	Traditional knowledge integration	Traditional knowledge workshops, consultations with elders, participatory mapping, and storytelling
	Disaster preparedness	Disaster risk assessments, consultations with experts, key informants, and community members
	Sustainable tourism development	Tourism surveys, key informants, participant observation
	Government-based adaptation	Community and government official consultations
	Adaptive infrastructure and technologies	Policy analysis, community meetings
	Capacity building	Personal experience, participant observation, and community consultations
	Community-based adaptation initiatives	Community meetings, participatory workshops
	Social capital and community ties	Social network analysis, community consultations, and consultations with elders

#### Data processing, analysis, and visualisation

Each indicator was normalised to a common scale to facilitate aggregation and comparison. The data for each year were processed to reflect cumulative values, with each subsequent year building on the previous total. Quantitative data were synthesised to examine trends in impact, vulnerability, adaptation, and resilience, offering a holistic view of the dynamics of human–environment relationships in the region. The qualitative data from interviews and ethnographic studies were transcribed verbatim and analysed using thematic analysis techniques. This process involved coding the data and identifying recurrent themes related to vulnerability, adaptive learning, adaptation actions, and resilience-building efforts.

Based on the qualitative and quantitative analyses, synthetic data were constructed to represent the identified trends. A stacked area chart was chosen as the visualisation tool to depict how each aspect (vulnerability, adaptive learning, adaptation, maladaptation, and resilience building) stacks up over the eight-year period to reflect the cumulative impact of climate change in Kashmir and the responses to it. To show the changes, yearly data points were plotted to observe trends over time. Colour coding was performed, in which each category was assigned a unique colour for clarity and visual differentiation. The Python programming language was used for data simulation and visualisation, while Matplotlib, a Python library, was used for creating the plot and ArcGIS Desktop 10.8.2 to create the study area map.

## RESULTS AND DISCUSSION

### Observed trends in climate impacts

The human dimensions of climate change in Kashmir are multifaceted. As temperatures rise and snow patterns shift, communities face different challenges. Disrupted livelihoods, health risks, and resource scarcity impact well-being, with a changing climate having direct and indirect impacts on livelihoods in Kashmir. Changes in precipitation patterns directly impact agriculture, particularly in areas dependent on rainfall for irrigation. Farmers reported reduced crop yields and increased vulnerability to droughts. Similarly, delays and shifts in snowfall patterns affect the livelihoods of those reliant on winter tourism, such as ski resorts and associated businesses. Both the precipitation and temperature have shown a change. The observations and experiences of the precipitation regime show that the overall precipitation (rain as well as snow) has decreased, and the precipitation pattern has changed, and the change is observed in terms of the shortage and deficit of rainfall and snowfall that have been experienced in the last eight years. In fact, in January, even if Kashmir did not receive snowfall, it used to receive rainfall, but that rainfall in January in the last eight years has shown a deficit, particularly in 2023 and 2024. Respondents across different age groups and socio-economic backgrounds have consistently perceived changes in temperature and precipitation over the past eight years. A significant proportion of the population reported experiencing warmer temperatures, decreased snowfall, and changes in rainfall patterns. Changes in climate have also had significant impacts on water resources in the region. Glacial retreat and less snowfall in the Himalayas have led to reduced water availability in the summer and autumn seasons for both domestic and agricultural purposes, resulting in increased competition and local disputes over water resources and water sharing. Climate change has also led to adverse health impacts, including heat-related illnesses and vector-borne diseases like dengue. It is essential to recognise that these impacts are not solely climate-driven but are also influenced by socio-economic factors like poverty, marginalisation, a lack of basic essential services, and inadequate healthcare infrastructure.

Kashmir relies heavily on glaciers for its water supply. However, these glaciers are receding, which disrupts the timing and volume of river flow, with implications for agriculture, hydropower production, and access to drinking water, potentially resulting in conflicts over water resources. Kashmir’s economy primarily relies on agriculture, with a substantial portion of the population engaged in farming and horticulture, growing fruits like apple, walnut, pear, cherry, peach, almond, apricot, pomegranate, plum, and mulberry. Climate change is altering temperature and precipitation patterns, which directly impact crop yields. As a result, agricultural productivity is declining, posing a threat to food security and livelihoods. Early flowering of horticulture plants has been experienced by the community, threatening the quality and quantity of fruits. A lot of people living near the Wular Lake depend on the lake for their livelihoods as they extract water chestnuts (locally called *Gaed/Gaer*) and fish, which have been threatened due to the drying up of a large portion of the lake. Climate change has affected the fishing community in Kashmir as community members struggle to catch a decent number of fish to earn their livelihoods due to the receding of water levels in streams and rivers.

Kashmir faces high vulnerability to disasters—such as floods, landslides, earthquakes, and avalanches—which have been exacerbated by climate change. It also results in a blockage of the main road that connects Kashmir with the rest of the world and a shortage of essential supplies. These events not only result in the loss of life and property but also disrupt livelihoods and infrastructure, further impoverishing communities. The increasing construction at tourist places in Kashmir like Pahalgam, Bangus Valley, Sonamarg, and Gulmarg is altering the natural landscape, contributing to environmental degradation, and threatening local ecosystems. Species of birds like the bulbul are not seen as frequently anymore. Beyond economic concerns, the cultural heritage of Kashmir is at risk. Traditional architecture, handicrafts, and traditional knowledge systems face challenges from extreme weather events, unpredictable and changing weather conditions, and habitat loss. Preserving cultural heritage is important not only for identity and tourism but also for maintaining community resilience in the face of environmental adversity. Saffron (locally known as Kong/Zaffron) cultivation in Pampore (Pulwama) faces challenges due to climate change, urbanisation, and industrialisation and has shown decrease in production and area under cultivation over the eight-year period, discouraging farmers from engaging in this activity.

Climate impacts are experienced by the people on a large scale (Table 2). An expert interviewee in 2024 narrated, “Out of round about 9500 glaciers in Himalayas, 95% glaciers are showing a receding trend. All the glaciers in Kashmir valley are showing a receding trend”. Community members are experiencing these impacts on their way of life, e.g. a community member in 2018 mentioned, “We have seen drastic changes in our livelihoods and lifestyle. The weather these days seems unpredictable and changing, and it devastates our crops most of the time”.

**Table 2:** Quotes describing how climate change impacts are experienced by the community members

Year	Quotes
2017	“We are perceiving the climate changes because of the variability that the climate is showing in Kashmir. We are having summers that are peaking at 37–38 °C now. This is the first-hand direct indicator of people experiencing climate change”.
2018	“Glaciers are melting so fast these days, which is not just a physical loss but also cultural and economic loss, affecting our ways of life”.
2019	“In the last few years, what we have experienced has been very little precipitation, both in the form of snow and rain. We could basically see that the whole Kashmir in the past was covered under snow by the end of December, and in January and February, the snow cover was at its maximum. But now it sometimes snows in late January or February”.
2020	“Water is a big challenge these days. We used to have plenty of fresh water, but we are feeling its scarcity over the last couple of years, which exacerbates existing social inequalities and disproportionately affects marginalised communities depending on agriculture”.
2021	“The drought-like conditions in the summers, when we need water the most for our crops, are affecting our livelihoods and coping mechanisms”.
2022	“Climate change has resulted in ecological changes that are reshaping our cultural and environmental landscapes and affecting our relationship with nature”.
2023	“Perceptions of climate change are interrelated with social and economic factors, affecting vulnerability levels and our adaptive capacities”.
2024	“Climate change is affecting our existence in this fragile Himalayan region. Although the conversation has shifted from denial to a call for social action, it is still not generally discussed”.

We examined whether observations had changed over time, specifically focusing on the magnitude, frequency, and severity of climate observations. Participants reported experiencing extreme heatwaves and cold waves, dry spells, untimely rainfall, delayed and less snowfall, and longer durations of drought like conditions, all of which were perceived as significant changes compared to past experiences. Observations of climate impacts in Kashmir have undergone notable changes over the past eight years. Initially, perceptions were predominantly centred around gradual shifts in temperature and precipitation patterns. Interviews conducted in 2017 indicated concerns about increasing temperatures during summers and erratic rainfall patterns, particularly affecting agriculture. A participant in 2017 remarked, “Earlier the seasons were quite predictable, but now we have seen differences in them, and they are unreliable. Summers are much hotter, and the rainfall is so unpredictable”. However, as the years progressed, the severity and magnitude of climate-related events have escalated. Data from climate records corroborate these observations, showing a steady increase in temperature and extreme weather events such as floods and droughts. By 2024, the frequency of extreme weather events had become a prominent feature of discussions with participants, who expressed heightened worry and anxiety about recurring floods, heat waves, and prolonged drought-like conditions disrupting livelihoods, water security, and exacerbating food insecurity.

The perception of climate change and its impacts among the local population has gradually evolved over time reflecting a complex interplay of impacts, adaptation, resilience, and growing concerns, but the change is not so profound. Initially, there was scepticism, lack of awareness, and a sense of disbelief and resignation, with some individuals attributing erratic weather patterns to their own sins and God’s punishment or natural variability rather than anthropogenic causes. However, recurring extreme weather events and scientific evidence have gradually changed people's perceptions. The perception has shifted from viewing changes as isolated incidents or God’s will to accepting them as part of a broader pattern of climate change. The general sentiment has evolved from denial to some understanding of the issues and a desire for adaptation strategies. Alongside the intensification of existing climate change impacts, new changes have emerged over the longitudinal period. Interviews conducted in 2017 revealed less concern about climate change. A respondent in 2017 mentioned, “It is all up to God. He will take care of the climate and everything. We don’t need to worry about it”. However, interviews in 2024 revealed growing concerns and a palpable sense of anxiety and frustration, coupled with a growing realisation of the need for collective efforts and actions. A respondent in 2024 reflected, “We need to realise that weather is changing a lot and should not deny it anymore. Climate change is real, and we need to act faster before we lose our habitat and everything”. A respondent in 2023 from a mountainous area expressed, “Our glaciers are shrinking and melting at a greater pace than ever. We rely on them for drinking water for ourselves as well as our animals and our crops, but they are disappearing so fast”.

These perceptions of climate change were highlighted by several participants, like; “Not only is this (rising summer temperatures, delayed and shifting precipitation patterns, low snowfall) the direct indicator of people perceiving climate change in the region, but there are other indicators like lack of icicle formation, negative impacts on livelihoods and traditional economic activities that you can understand that the climate in the region is changing”. This interviewee in 2018 highlights the direct indicators of climate change that people in the region are perceiving. “The winters have been warming very much, and then they're followed by the spring. There is a little bit change in the autumn, but summers are also showing the trend, that's increasing one. And both mean, I mean, average temperatures, the maximum temperatures and the minimum temperatures are witnessing the rise”. (Interviewee, 2019). “But the concern is about the minimum temperatures in the winter that have actually gone up, so that will have a drastic consequence in terms of the winter precipitation, that is, our snowfall, that will not actually turn into ice and then can sustain our streams in the subsequent months. So, that is what we are experiencing currently and will be a challenge in the future” (Interviewee, 2020). A noticeable change has been experienced by people over the years. “What we have seen in the last 50 years is that both the precipitation and temperature in Kashmir have shown a change. Precipitation regime shows a shortage of rainfall and snowfall in the last 30 years”. (Interviewee, 2021).

The study explores how people's feelings towards climate change have evolved over the eight-year period. Initially, participants expressed concern about the changes they were witnessing, but there was also a sentiment of denial. However, as the study progressed, it became evident that the community members began to accept the changes as unavoidable and to consider adaptation strategies to deal with the effects of climate change. This shift in attitude was related to the rising frequency and severity of climate catastrophes, which affected everyday lives and livelihoods. In order to reduce the negative effects of climate change in Kashmir, participants recognised the necessity of collective action and adaptation measures. People began to demand more accountability from policymakers, as well as increased measures to mitigate climate change and build resilience. However, the importance of addressing climate change is still not a concern for the majority of the population.

Kashmir used to have a clear distinction between the four seasons. Winter typically lasts from December to February, spring lasts from March to May, summer lasts from June to August, and autumn typically lasts from September to November. But climate change has blurred the distinction between seasons. An expert mentioned in 2024, “In terms of four distinct seasons, what we have been experiencing for the last few years is that, as far as this core winter or the winter is concerned, it is getting shortened. So that means when it is getting shortened, you have early spring, and early spring is the duration of winter basically that is shortened”. People often remark that the weather feels like the spring season weather, even during the winter months, and sometimes winter-like conditions extend to the spring season, as seen in 2024, where Kashmir experienced continuous rains accompanied by hailstorms in March and April. This shift in seasons impacts daily life, agriculture, and overall well-being.

In Kashmir, there has been an increase in the frequency of delayed snowfall in the last few years. The coldest forty-day period in Kashmir typically starts around December 21 and ends on January 29, which is locally known as “Chillai Kalan”, and most of the snowfall takes place during this period. The high amount of snowfall during this period helps in retaining the snow on glaciers for a longer duration, which then helps in ample water availability throughout the year. However, reduced and delayed snowfall during Chillai Kalan has been experienced. It was particularly evident in the winter of 2024, when there was almost no snowfall in the whole Chillai Kalan in the plain areas. This has resulted in the irrigation of horticultural fields by farmers for the first time during the winter season. Special prayers were organised at several places for snowfall to occur. These types of prayers (called Bandaars) have a special place in the culture of Kashmir, as sometimes when there is a scarcity of rainfall or snowfall or there is a disaster or excess rainfall, people organise these prayers. Due to the absence of snowfall in winter, winter tourism at places like Gulmarg (turning the tourist place into cricket fields), where thousands of people visit for skiing, is affected. This leads to cancellations of tickets and hotel accommodations, affecting the businesses and livelihoods of communities, as evidenced in 2024. Dry winter spells have affected the footwear business, resulting in unsold stocks of winter waterproof shoes and a reduction in sales. Thus, climate change is affecting the key economic sectors in Kashmir.

Perceptions regarding the changing climate are corroborated by recent data. In January 2024, the Kashmir region experienced unusually high temperatures, marking it as the warmest January on record, with Srinagar experiencing its sixth warmest January day in 133 years on January 13, reaching a temperature of 15 °C. The absence of snowfall and the inactivity of Western Disturbances contributed to exceptionally high temperatures, surpassing normal levels by more than 8 °C on some days during the month. Srinagar recorded its warmest-ever average maximum temperature for January at 11.7 °C. Additionally, other regions in Kashmir, including Gulmarg, Pahalgam, Qazigund, Kokernag, and Kupwara, witnessed their highest-ever winter temperatures. For instance, Gulmarg recorded a mean maximum temperature of 5.4 °C, Pahalgam 10.3 °C, Qazigund 12.1 °C, Kokernag 10.9 °C, and Kupwara 11.6 °C (IMD Srinagar, 2024; The Kashmiriyat 2024; Gillani 2024). These observations are supported by people's experiences regarding changes in the timing of seasons. People often remark that the weather feels like March, even during the winter months. “It feels like *marchuk taaf* (March like temperature). You can see there is no snowfall. The sun is so bright. I haven’t seen such a thing in my life when there was no snowfall in Chillai Kalan. We used to play on snow, and I miss those days”.—Participant, January 2024.

On 28 July 2024, Srinagar experienced its fourth highest temperature of 36.2 °C, Qazigund experienced its highest ever temperature of 35.6 °C, and Kokernag experienced its highest ever temperature of 34.1 °C throughout the history. While Qazigund recorded a temperature of 32.6 °C, Srinagar recorded its hottest May day in the last decade on 23 May 2024, with a temperature of 32.2 °C (IMD, 2024), surpassing the previous record set in 2013. Kashmir witnessed heat waves in 2024, which caused discomfort among the population, disruption of daily life, a water shortage, and protests demanding adequate water supply. These heat waves occurred during the period when people started irrigating their crops and kitchen gardens, thus increasing the demand for water. These heat waves were followed by cloud bursts and flash floods at a few places.

Experts on climate change interviewed as part of this study are of the view that over the last 50 years there has been a noticeable change in the climate, particularly in the last 30 years. Temperature and precipitation, specifically snowfall and rainfall, have decreased in the region. This decrease in precipitation has led to a shortage of water resources, which is crucial for agriculture, hydroelectric power, and the tourism industry. Glaciers are receding at an alarming rate, with 95% of glaciers in the Himalayas showing a receding trend, including those in Kashmir. This loss of glaciers not only affects water availability but also has long-term consequences for the ecosystem and biodiversity. The decrease in snowfall has affected the length of the winter season, leading to early springs and shortened periods of snow cover. This has implications for agriculture as well as the overall climate patterns in the region. These changes in Kashmir's climate are a result of global warming, urbanisation, pollution, and other factors such as weak westerlies and the El Niño effect. The decreasing snowfall, combined with increasing temperatures, has affected water availability, hydroelectric power generation, and the tourism industry.

### Gendered impacts of climate change

Women in Kashmir are disproportionately affected by the impacts of climate change due to a range of social, economic, political, and cultural factors. These factors include limited access to resources, education, and decision-making power, as well as patriarchy and traditional gender roles and responsibilities. Women's vulnerability is further exacerbated by their roles as primary caregivers and their reliance on climate-sensitive sectors such as agriculture. These impacts are particularly prevalent in rural areas and marginalised communities.

In the context of the marginalised Gujjar community, a pastoralist community that predominantly inhabits mountainous regions across the Kashmir Himalayas, the gendered impacts of climate change are particularly significant. Their livelihoods are intricately tied to the natural environment, relying on livestock rearing, agriculture, and forest resources. Climate change disproportionately affects them due to rising temperatures, erratic precipitation patterns, and glacial melt. These changes directly impact their traditional way of life, exacerbating existing vulnerabilities. Water scarcity is a critical issue in their habitats. Glacial retreat reduces water availability, affecting both humans and livestock. Gujjar women play a pivotal role in water accessibility. They are responsible for fetching water from distant sources, often trekking long distances.

The burden of water collection falls disproportionately on women due to cultural norms and traditional gender roles. This is experienced by women in different parts of Kashmir, as due to the scarcity of water, they travel longer distances to fetch water for drinking and irrigating their vegetable gardens, as gender roles dictate their responsibility for cooking and vegetable farming. This has resulted in several protests by women across Kashmir against water scarcity. Lack of safely managed water and sanitation services affects women and girls significantly. They often spend hours fetching water, impacting their time for personal well-being, work, and education (Table 3). Women wake up early in the morning to prepare Kangris (a Kangri/Kanged is a clay pot wrapped in wicker and filled with hot embers that serves as a personal heater to stay warm during chilly weather) and hot Kashmiri salt tea (Noon Chai) during the cold winter seasons, which is physically demanding, leading to frostbite and cough. Some women wash clothes in cold water in winters, sometimes at home and sometimes at rivers, due to the unavailability of hot water and washing machines in their homes, which affects their health as the cold weather makes tasks like washing, chopping, and cooking more challenging due to discomfort of low temperatures and stiff fingers. The gendered division of labour assigns women the responsibility of maintaining cleanliness and hygiene within the household. The lack of hot water availability reinforces existing gender roles, where women are expected to manage laundry even under challenging conditions. As climate change intensifies, the gendered impact of water scarcity becomes more pronounced. The seemingly mundane act of washing clothes in cold water reflects broader gender inequalities, disparities in access to basic amenities and climate challenges. Climate change is further exacerbating the patriarchal norms and marginalisation of women, leading to inequity and gender disparity, making it imperative to understand gendering water and climate change.

**Table 3:** Quotes highlighting the gendered impact of climate change

Year	Quotes
2017	“Winters are harsh in Kashmir, and the patterns are changing. Sometimes they are a little warm, and sometimes very cold, which is uneven. It didn't happen before. I do all the household work, so normally what I do in the winter is I get wood from the forest for heating and cooking”.
2018	“Recently due to drying of ponds we don't get water in our houses, so what I do is travel at least two to three kilometres down to the streams to get water. I do it every day and bring it on my head, sometimes twice or thrice a day, and we don't have all the facilities at home”.
2019	“We see the kind of patriarchal society we live in, where women have to do most of the household work. Even during my pregnancy, I still had to go to get water and cook all the time”.
2020	“ We see so many women protesting for water, especially in the summer, because there's no water in some areas as the rivers are dry. So, we don't get water in summers, and this water scarcity is obviously putting a lot of pressure on women because, normally, you will see men are not taking part in these activities. So, it's the women who have to go down the streams to get water”.
2021	“ Even in the winter, when the temperature is negative, it's the women who have to walk down these rivers in the villages to get water, and also firewood”.
2022	“ It hard for women to access healthcare, education, and economic opportunities. The nearest hospital is 10 kms away ”
2023	“Climate change is affecting women harder because our husbands are mostly labourers, and during the winter they hardly get any work, so it's the women who have to take care of everyday life”.
2024	“ Being a mother, I am concerned about my children's health and future as the climate change worsens. We need to have gender-responsive policies to enable empowerment of women”.

The gendered impacts of climate change documented in this study include:

i. Physical labour and health risks: Women engage in arduous physical labour while carrying heavy water containers across mountains, hills, and rugged terrains, even during heavy rainfall, snowfall, and high temperature. This impacts their health and well-being, e.g. a participant in 2018 mentioned, “It takes me one hour to go to get water and then one and half hour to get back, climbing the steep mountain slope. I carry the big container on my head. This has weakened my legs. I feel so tired when I climb the mountain with the water container, and then I have to cook for my family”. Women are at risk of health problems due to exposure to extreme weather conditions during water collection, e.g., a participant in 2017 narrated, “I broke my leg while walking with a water container”.
ii. Time constraints: Women's limited availability for education and economic generation is a direct result of the time they spend cooking, collecting water, and vegetable farming, e.g. a girl in 2022 narrated, “Seeing my mother do all the household activities makes me feel sad. I then help my mother in collecting firewood, feed the cattle, and cook, so I don’t get time to go to a school. Also, the school is too far from here, almost 5 kms. I can’t walk so much everyday”.
iii. Empowerment: Patriarchal norms and limited access to economic opportunities, education, and other resources hinder the empowerment of women, which often translates to power within communities, e.g. a participant in 2021 mentioned, “I have to ask my husband to give me some money. I don’t earn myself; I just cook and do household chores. Even I don’t have money at times to buy sanitary pads”. Another participant in 2022 mentioned, “what kind of life is it…I mean if a woman works, she is looked down with as if she has committed a sin”.

### Vulnerability trends

Vulnerability to climate change in Kashmir was observed to be increasing based on observations of community members over the 8-year period, driven by environmental degradation, socio-economic factors, and insufficient policy measures. Climate change has exacerbated existing vulnerabilities in Kashmir. Socially marginalised groups are disproportionately affected, and disruptions in agriculture, water availability, and livelihoods enhance vulnerability. For instance, farmers face crop losses due to erratic weather patterns. Communities’ social fabric is strained as they adapt to changing conditions. The past eight years have seen an increase in vulnerability because of various factors like disasters, changes in land use, socio-economic problems, the loss of agricultural land to horticulture and settlements, the blocking of floodplains, which increases the risk of flooding, and the receding of glaciers, which increases exposure to risk. These factors are further compounded by noncompliance with building codes and guidelines. A lot of people live in the flood plains, which are highly vulnerable to recurrent floods in the Jhelum River. Women and marginalised communities in Kashmir like the tribal communities of Gujjars (pastoral community) and Bakarwals (who migrate seasonally with their cattle), Waatals, and Hanjis (who live on water in houseboats) are the most vulnerable to the impacts of climate change.

**Fig. 2:**
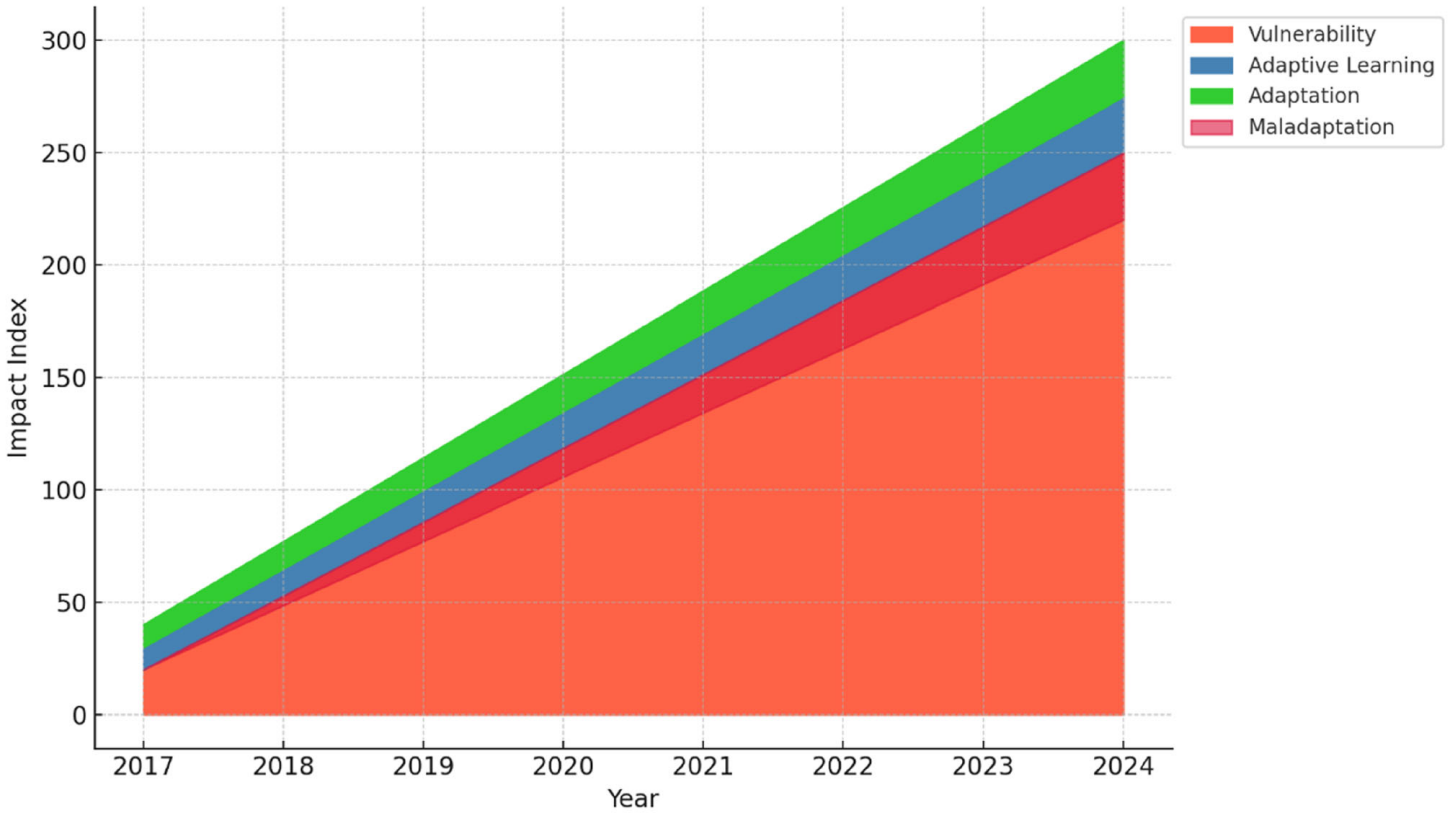
Climate change scenario in Kashmir

**Fig. 3:**
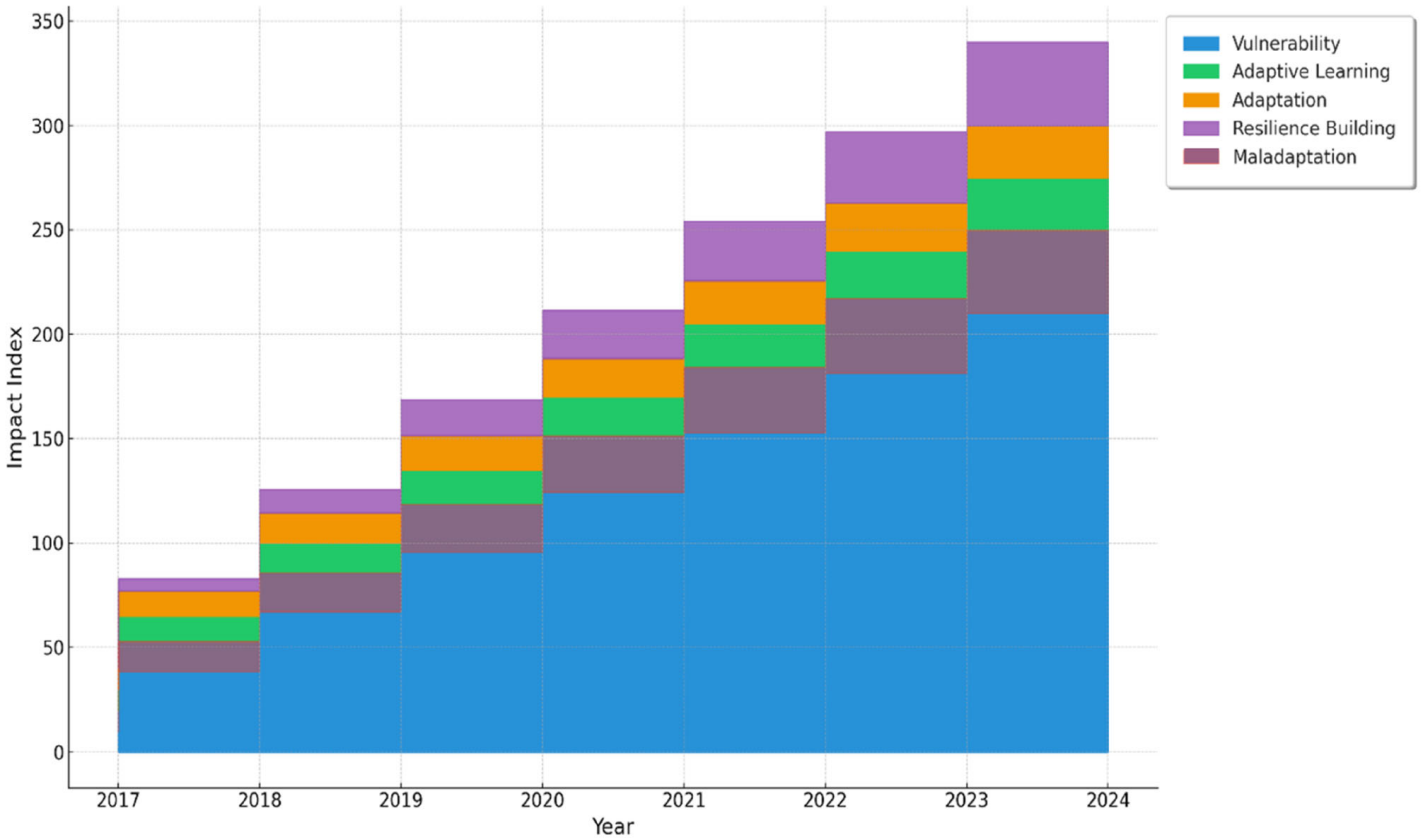
Climate change impacts and response trends in Kashmir

The findings of this longitudinal study indicate that vulnerability in Kashmir has undergone substantial changes (Table 4, Figs. 2, and 3). The region has seen an expansion in unplanned urbanisation, particularly in low-lying floodplain, exposing residents to a variety of risks. The encroachment on wetlands and water bodies, combined with deforestation, has led to an increased risk of flooding, landslides, and avalanches. Temperature and precipitation patterns in the Kashmir Himalayas are changing, as is the rate of glacial retreat, affecting the availability and timing of water resources. This has serious ramifications for the sustainability of livelihoods, hydropower production, and agriculture.

**Table 4:** Quotes describing vulnerability trends

Year	Quotes
2017	“We have seen an increase in the loss due to disasters, but this loss is more on vulnerable people who have limited resources to cope with the loss”.
2018	“As a poor person living in an urban area, I have seen increasing pollution and heatwaves. I can’t afford to get an air conditioner. Earlier we didn’t need one”.
2019	“ In the last few years, we have experienced increasing social inequalities and the plight of the most vulnerable sections of society”.
2020	“Changing climate is further creating vulnerabilities, particularly for farmers and households headed by women who face hurdles in accessing water, food, and shelter during disasters”.
2021	“Seeing more floods along the Jhelum River, we migrated but we don’t have good facilities like transportation, drinking water and hospitals here”.
2022	“ Our whole life depends on agriculture, but it is affected a lot due to changing weather conditions and creates economic vulnerabilities and poverty”.
2023	“ I have seen a lot of people crying who are engaged in horticulture because most of their produce was destroyed by rain and hailstorms. It was all they had”.
2024	“Over the last eight years, the vulnerability of people to climate-induced disasters has increased. A lot of times the rainfall is for a short period and the amount of rainfall received is huge, but overall, the amount of precipitation has decreased. These brief spells of heavy downpours basically initiate floods and landslides, so the vulnerability of the people living in these areas has increased. We have seen terrible heat waves this year”.

On the social dimension of vulnerability, the region lacks adequate access to basic services such as clean water, sanitation, electricity, and healthcare. Climate change, limited resources, restricted mobility, and increased risk exposure have increased the vulnerability of women and marginalised communities. Economic vulnerabilities have evolved because of the substantial reliance on climate-sensitive sectors like agriculture and tourism, and patriarchal norms. Fluctuations in agricultural production have resulted in income variability and increased livelihood insecurity. Institutional vulnerabilities, such as weak governance structures and inadequate disaster preparedness mechanisms, have exacerbated the climate crisis. Lack of coordinated initiatives, effective policies, and institutional capability has hampered vulnerability reduction and response strategies.

The observed climate change impacts, such as temperature increase and precipitation pattern shifts, affect the vulnerability of the communities, particularly farmers, as a respondent in 2018 narrated, “our crops are increasingly becoming vulnerable to weather conditions. Last year, the hailstorm destroyed my apple orchard. My whole family depends on rice and apple cultivation. Due to incessant rains, I incurred a loss of hundreds of thousands of rupees”. While describing the power dynamics of the apple cultivation a respondent in 2021 narrated, “I had taken a loan for my daughter’s wedding from a local apple dealer in the hope of returning it from the profit I get from apple cultivation. But this year the production was so low due to changing weather conditions that I couldn’t pay him back. Now he is asking to sell him the apples next year with less price”. An expert in 2023 while describing the disaster vulnerability in the region mentioned, “vulnerability of people to different types of disasters has increased…landslides, floods, and other disasters have increased, which has increased vulnerability of people living in these areas". The lack of awareness and understanding further contributes to their vulnerability.

"We traditionally call it attitudinal vulnerability. We are witnessing it, but we are not going to adapt to the change. We are not going to adapt to the transformative practices or even the reactive practices… People are less concerned about it. People prefer to see Kashmir as a tourist place and so people are after the kind of the activities that will fetch them more and more profit" (Interviewee, 2024). This quote highlights the attitudinal vulnerability and resistance to adaptation among the population in Kashmir. It shows that the pursuit of economic gain, particularly in the tourism and horticulture sectors, hinders efforts to address climate change and prioritise resilience-building measures.

### Differential vulnerability

Our study analyses the differential vulnerability to climate change in Kashmir, drawing upon examples of Gujjar, Bakarwal, and Waatal communities. They serve as prime examples of how structural inequalities, deeply embedded in economic, political, and cultural contexts, intersect with the impacts of climate change.

#### I) Gujjar and Bakarwal communities

Gujjar community in Kashmir usually practises agriculture, rears buffaloes and cows, and resides mostly in mountainous areas of the Kashmir Himalayas, which are vulnerable to flash floods, landslides, and snow avalanches. Some of the community members practise transhumance. Although the Gujjar and Bakarwal communities have some similarities, they are both two different communities. Bakarwal community is a sheep and goat rearing transhumance community migrating to highland pastures in Kashmir Himalayas during summer (locally called *nyor*) and low-lying plains, usually outside Kashmir, in Jammu, in winter along with their cattle. Their livelihoods depend on grazing pastures and forests, and selling their cattle for economic returns. These grasslands have shrunk due to climate change, encroachment, and reduced access due to different forest conservation and protection policies. The changing weather patterns, like the incessant rain and lack of rain when required for the grasslands, have affected the community and resulted in the deaths of cattle on multiple occasions. As mentioned by a Bakarwal in 2018, “Ten of my cattle died because of the incessant rains. Two sheep fell from the mountain and died, and I didn’t get any compensation". A Gujjar in 2023 narrated, “Three of my cows were eaten by a bear, as we see a lot of bears coming down to our places due to the cutting down of the forests”. Both communities travel long distances to access water, firewood, and educational and healthcare facilities, which are often lacking. They have historically faced systemic discrimination and marginalisation. Racism is so common that there is an infamous saying in Kashmir: “If you see a snake and a Gujjar in a forest, kill the Gujjar and spare the snake because Gujjars are more dangerous than snakes. Deception is in their blood”. They are called derogatory terms like Gujir Nasal (Gujjar race), Bakarwal Nasal (Bakarwal race), Gujir Fakh (Gujjar smell), Bakarwal zaat (Bakarwal caste), Gujir and Bakarwal Khaslat (characteristics), Gujjir mass (long hair like a Gujjar), and Gujjur huv (looking like a Gujjar), which have been normalised in everyday conversations. The attitude towards these communities has not changed much over the years, and they continue to be marginalised.

The vulnerability of Gujjar and Bakarwal communities documented in this study include:

- Discrimination, dehumanisation, racism, and othering: The continued existence of discrimination against the Gujjar and Bakarwal communities can be understood in the larger historical and socio-political context of Kashmir. These communities which have been historically excluded experience oppression as a result of deeply rooted power relations and socio-economic inequities. Both communities experience dual discrimination rooted in both their occupation and ethnicity as a significant proportion of them are predominantly nomadic herders. This intersectionality intensifies their marginalisation, as they are often perceived as outsiders by mainstream society. This form of alienation perpetuates detrimental perceptions and rationalises acts of violence or indifference towards them.
- Medical neglect as a form of structural racism: The act of refusing medical treatment due to biassed judgements of odour exemplifies structural racism within healthcare systems. For example, in 2017, a woman from the remote Moore village in Kupwara district encountered medical negligence when doctors at Lal Ded Hospital in Srinagar (the capital city of Kashmir) refused immediate treatment. Their biassed justification is expressed as “amis chu fakh yiwan, yim chi Gujjir” (they smell foul, they are Gujjar). Despite the intervention of another doctor, the woman was denied an overnight stay and gave birth on the road, with the newborn dying (Yasir 2024). Discriminatory attitudes among healthcare professionals cause unequal access to healthcare services for them. It violates their fundamental rights, perpetuates health care disparities, and exacerbates existing inequalities. The lack of veterinary facilities and doctors leads to disease and death of their cattle.
- Language as a tool of oppression: The casual use of the terms "Gujjar" or "Bakarwal" and "Gujir Nasal", Bakarwal Nasal”, "Gujir Khaslat", “Bakarwal Fakh”, and "Gujir Fakh" as derogatory language in ordinary discussions highlights the normalisation of discrimination and racism in Kashmiri society and demonstrates how language is used as a tool of oppression to reinforce stereotypes and justify discrimination. These terms dehumanise both communities by reducing their cultures to essentialised traits connected to undesirable qualities.

The convergence of discrimination, disparity, and marginalisation exacerbates the vulnerability of Gujjar and Bakarwal communities to climate change impacts by limiting their access to resources, widening economic gaps, excluding them from decision-making processes, increasing their exposure to environmental risks, compromising their health, and disrupting their cultural practices. Climate change and a lack of basic facilities have forced some members of both communities to abandon their traditional livelihood practices and engage in manual labour in the construction of houses and agriculture. Bakarwals are a nomadic community and do not possess their own land. Their whole life depends on cattle, sheep, poultry, and horses, which is their wealth. Climate change is exacerbating their vulnerability by affecting grasslands, which is affecting their livelihoods and threatening their existence. Rooted in historical marginalisation and power dynamics, the injustices underscore the need to challenge ingrained attitudes, dismantle systemic barriers, and promote social justice for marginalised communities in Kashmir. The Gujjar and Bakarwal communities are very resilient and take pride in their identity, and their resilience has stood time and again against continued marginalisation, discrimination, and climate change.

#### II) Waatal community

The term “Waatal”, also known as Mochi or Sheikh, means cobbler in the Kashmiri language. Waatal community is facing issues like occupational stigma, marginalisation, underreporting, endogamy, social isolation, and religious and cultural discrimination. Traditionally, the community has been associated with occupations such as cobbling, tanning and scavenging. These professions were considered “polluting” or “low status” by other communities. Consequently, the Waatals faced social exclusion and were sometimes restricted from entering temples and shrines. According to the 2001 Census of India, the Waatal population was recorded as a mere 169 people (Census of India, 2001). However, this number is likely lower than the actual count because of the stigma attached to the community’s identity. Fear of prejudice and discrimination may have prevented many Waatals from accurately self-identifying. Endogamy is practised in the community. While this helps maintain their distinct social and cultural identity, it also leads to isolation from other communities, who do not usually marry them. People from other communities did not use to attend their weddings with the prejudice of food being "polluted," washing the dishes "thoroughly”, or completely throwing them away if touched by a Waatal. People often use racial slurs like *waatul huv chukh* (looking like a waatul) for a dark-skinned person, *waatal khaslat* (traits like a waatul) for rude behaviour, and *sraan kerziha, zan chukh waatul* (you should bathe; you look like a waatul) for an untidy person. This reflects the deep-seated racism within Kashmiri society. Social interactions and opportunities for upward mobility are limited. Some of the discriminatory practices have decreased over time, but prejudice and stereotypes persist.

The Waatal community has historically engaged in occupations like manual scavenging, cobbling, tanning, and sweeping. These professions often involve working outside and being exposed to pollution and environmental conditions. As climate change intensifies, extreme weather events such as heatwaves, cold waves, heavy rainfall, and flooding affect their socio-economic activities. The combination of low socio-economic status, increased health risks, discomfort from extreme temperatures, and marginalisation makes them highly vulnerable. Waatals who rely on water for tanning and other activities, may experience water scarcity due to climate change. Reduced water availability impacts their livelihoods and exacerbates existing challenges. While working in open spaces, they are at risk of heat exhaustion, dehydration, heatstroke, and cold. Waatals face the need to migrate due to changing weather patterns, loss of livelihoods, and disasters like floods, as a lot of the community members live along the riverbanks and floodplains. They have historically encountered inequality, discrimination, and limited access to resources, which are exacerbated by climate change, e.g. a participant in 2017 mentioned, “I was denied flood relief just because I belonged to the Waatal community. The person in charge kept delaying the payment. We are discriminated against at every level". One participant in 2019 narrated, "The floods have damaged our house as well as cattle, and we have experienced increasing instances of floods in recent years. I think it has something to do with changing weather conditions”. Another participant in 2023 mentioned, “Even natural calamities don’t spare us. It seems they especially target poor people like us. We live on a riverbank. We might be forced to leave this place if such conditions continue to exist”.

The Waatal community’s resilience is tested as they adapt to changing environmental conditions. Access to resources for adaptation, such as better housing, healthcare, education, and social protection programmes, remains limited. Climate change threatens their traditions and cultural practices. Recognising the specific vulnerabilities that marginalised communities, such as the Waatal community, face in addressing climate change is necessary, and measures to maintain their cultural resilience should be incorporated into climate action plans.

### Adaptation and double-edged sword of maladaptation

Communities’ attempts at adaptive learning and adaptation have made minimal progress, despite the increasing vulnerability. Constraints to adaptation include limited awareness programmes, slow adoption of sustainable practices, and inadequate implementation of adaptation policies. A varied response to climate change adaptation was found in Kashmir when policy and institutional frameworks were analysed. Even though there have been some initiatives—like creating climate change departments and creating action plans—effective adaptation measures are hampered by implementation gaps, lack of funding, and community-based adaptation initiatives. Weak coordination among government agencies, inadequate funding, and poor implementation of climate change adaptation plans were noted as key challenges.

Ad hoc measures, such as shifting from paddy cultivation to horticulture, aim to adapt to changing conditions, but new crop varieties requiring more water highlight the need for improved irrigation facilities. These changes affect agricultural practices and livelihoods, leading to shifts from traditional agriculture to horticulture. However, adaptation efforts are limited and hindered by a lack of awareness and understanding among the general population, as well as inadequate policy frameworks and implementation.

Over the past eight years, Kashmir has witnessed notable adaptations across various sectors (Table 5). A lot of people have started getting involved in the tourism sector to establish their travel and tour facilities. Agricultural changes include the adoption of new techniques and crop varieties like the hybrid varieties of apples and vegetables. Education has seen a move towards adopting more courses on disaster management, particularly in colleges. Some farmers have started diversifying their livelihoods, such as through new apple varieties, indoor saffron cultivation, musk melon, strawberry, and mushroom cultivation, and some government schemes aim to support this diversification. A farmer in 2023 noted, "We've had to adjust our farming methods to plant new varieties that grow fast and provide more profit". Similarly, a tourism operator in 2021 remarked, "I lost my job during the COVID-19 pandemic, and then I established by tour and travel agency, and I am doing okay with my earnings". However, there are challenges and potential maladaptation in such shifts, with some farmers facing losses and difficulties in adopting new varieties or practices and the cost of transitioning to horticulture.

**Table 5:** Quotes describing adaptation efforts

Year	Quotes
2017	“Following the loss of my business due to floods, I got a cab and take tourists to different places”.
2018	“Due to water scarcity, I switched to drip irrigation".
2019	“We installed solar panel to cope with electricity shortage during extreme weather events”.
2020	“Our school has initiated tree planting campaigns to curb deforestation”.
2021	“During COVID-19 and recent floods, our village established community-based relief initiatives".
2022	“To save our water bodies, we revived traditional community-dredging initiatives through traditional knowledge”.
2023	“ To diversify my livelihood, I have started growing mushrooms and strawberry”.
2024	“Having realised how crucial education is to fostering resilience, I've begun educating my kids about sustainable living and climate change. Knowledge transfer is the key to preparing our future generations to adapt to climate change".

Participants emphasise that people are not adapting sufficiently to the changing climate and prioritise personal gains over long-term sustainability. They emphasise the importance for a balanced approach to adaptation and suggest adopting traditional knowledge, ecosystem-based approaches and practicing a community-led, collaborative effort to improve resilience. The participants, when asked about coping strategies and adaptation, remark that some people have switched to high-density apple varieties for higher profits but acknowledge that there are complications and risks involved. They observe a lack of proactive, transformative adaptation and emphasise the importance of bottom-up resilience building. Resilience building initiatives have reportedly been minimal.

The lack of a strong framework for assessing adaptation and vulnerability hinders the region's ability to effectively address climate change impacts. This lack of understanding inhibits the deployment of effective adaptation measures. Despite the community’s vulnerability and the observed impacts of climate change, there is a lack of understanding, awareness, and an effective framework for adaptation. Although the government's programmes and subsidies are intended to promote adaptation, they are not adequately guided by scientific research and local knowledge.

The study challenges the notion of adaptation and resilience in the context of climate change in Kashmir and asserts that the concept of adaptability is class-centric, and the most vulnerable communities lack the resources and technology necessary for effective adaptation. The shift from paddy rice cultivation to horticulture is due to economic factors rather than climate change considerations. The study challenges the dominant discourse on climate change, emphasising the need for a localised understanding of vulnerability and adaptability.

Maladaptation has emerged as a significant concern, with certain adaptation strategies inadvertently exacerbating vulnerability, highlighting the need for more nuanced and sustainable approaches. Instances of maladaptation have inadvertently exacerbated the vulnerability of certain communities. For example, the introduction of cash crops and the increasing shift to horticulture as a quick adaptation measure to diversify livelihoods have led to increased water usage and soil degradation, as a respondent in 2023 mentioned, “I cut down the traditional apple varieties and converted my paddy land to horticulture, but I need to water these new varieties regularly, but there is a shortage of water here”. Another respondent in 2021 mentioned, “I need to use more fertilisers, which has decreased the natural fertility of my land. Every year, I use more and more fertilisers”. These examples highlight the need for a more nuanced understanding of adaptation impacts. Some well-meaning adaptation efforts have had adverse outcomes. This was seen when flood barriers were built, inadvertently diverting floodwaters to vulnerable settlements, and increasing their vulnerability. An increase in the use of *hamams* (a traditional heating system used in homes by burning wood) and *coal bukharis* (a type of stove that burns coal to produce heat) has been experienced, leading to higher smoke concentrations, air pollution, and deforestation. These examples of maladaptation demonstrate  the difficulty of applying adaptation strategies without having a thorough understanding of the local socio-ecological systems.

### Climate change is not a priority/main concern

Climate change is not seen as a priority for many people in Kashmir, as their main concerns are focused on politics and conflict. Conflict takes a lot of space in people's minds as well as public spaces. Although climate change impacts have become more noticeable and experienced on an increasing scale over the eight years of observation, people have not become more concerned or seen it as more of a priority. The limited discourse of climate change among the general population in Kashmir is influenced by other pressing concerns, such as conflict, security, economic insecurity, unemployment, poverty, political instability, lack of basic facilities, welfare, disease, and misery. Lack of anthropogenic consciousness around climate change exists and the attention and discourse on climate change is limited to a niche of scholars and researchers who work directly or indirectly on the topic. These scholars focus primarily on quantitative aspects rather than the broader social impact. The marginalised communities in Kashmir are more vulnerable to the impacts of climate change but still there is limited awareness and understanding of these issues among the wider population due to political disturbance. While vulnerabilities are increasing in the region, the perception and response to climate change are shaped by various socio-political factors, including the ongoing conflict and political instability. India, Pakistan, and China have been involved in a political battle over Kashmir for almost eight decades. Climate change and other environmental issues are frequently overshadowed by the region's persistent violence and political instability. Policymakers often prioritise these issues over environmental concerns.

The quotes in Table 6 illustrate the predominant belief that climate change is not a priority in Kashmir given the ongoing political turmoil. Nevertheless, there are signs of a possible change in mindset throughout the 8-year period of study, as certain community members increasingly voice apprehension to climate change. This emphasises the necessity of adopting comprehensive approaches that address both environmental and societal problems in Kashmir.

**Table 6:** Quotes on climate change priority

Year	Quotes on climate change not being a priority	Changes over time
2017	“We are more concerned about our safety, security and jobs than climate change. The immediate concern for me and my family is our survival”.	–
2018	“I am a poor person; I don’t have a regular job. I am a manual labourer, and sometimes it is hard to find work. I have to feed six members of my family. This is my immediate concern, not the changing weather”.	–
2019	“Our minds are occupied by politics and conflict, which have become part of our daily lives and conversations, and thinking about climate change is a luxury”.	–
2020	“Survival in the present takes precedence over climate threats. We will address climate change when our basic issues are resolved”.	–
2021	“With all the political unrest around us, climate change looks like a far-off menace. Our priorities right now are stability and security”.	–
2022	“Climate change is beginning to garner prominence in our discourse, primarily because of the recent floods, but political tensions continue to overshadow it”.	Some community members show increasing concern as the effects of climate change become more apparent
2023	“Although the seriousness of climate change is becoming more apparent, it still contends with recurrent economic and political crises for public attention”.	More people understand the need to address climate change, but for many, it still comes after more pressing political issues
2024	“We are increasingly recognising the interconnectedness of recent heat waves and climate change with our social, political, and economic problems. Maybe it is time to give political and climate concerns equal priority”.	A slight transition is occurring as certain community members acknowledge the interdependence of climate change and larger societal problems, while it remains of little concern for many

## Conclusion

Our longitudinal re-study of climate change vulnerability in Kashmir tracks changes and developments over the past decade and reveals a complex interplay of environmental, social, and political dynamics and the conspicuous absence of a critical issue from the political discourse: the preservation of the Himalayas. Our Himalayan Re-study Framework (HRF) is significant to understand climate change vulnerability not only in the Kashmir region but also in other Himalayan regions and at the global level. The impacts of climate change are increasingly evident in Kashmir, with rising temperatures, erratic weather patterns, and extreme events affecting communities' livelihoods, health, and well-being. Local observations and personal experiences highlight the differential vulnerabilities faced by marginalised groups, women, and those dependent on agriculture and natural resources. Despite the growing recognition of climate change impacts, they remain overshadowed by more immediate concerns, such as political instability and economic opportunities. Climate change in Kashmir cannot be understood without taking politics into consideration. Addressing climate change vulnerability in Kashmir requires integrated approaches that recognise the complex interactions between environmental, social, and political factors. Adaptation and building resilience necessitate empowering communities, social justice, equity, fostering inclusive decision-making processes, and a gender-sensitive and inclusive approach that empowers women and marginalised communities and promotes their active participation in climate action.

## Data Availability

Data are included within the manuscript.
